# Accelerated partial breast irradiation using once-daily fractionation: analysis of 312 cases with four years median follow-up

**DOI:** 10.1186/1748-717X-7-17

**Published:** 2012-02-06

**Authors:** Arif Y Shaikh, Michael A LaCombe, Hongyan Du, Vathsala T Raghavan, Ranjeev K Nanda, William D Bloomer

**Affiliations:** 1 Department of Radiation Medicine, NorthShore University HealthSystem, 2650 Ridge Ave. Evanston, IL 60201, USA; 2 Center for Clinical and Research Informatics, NorthShore University HealthSystem, 2650 Ridge Ave. Evanston, IL 60201, USA

**Keywords:** breast, APBI, IMRT, DCIS, invasive lobular carcinoma

## Abstract

**Background:**

There are limited data on accelerated partial breast irradiation (APBI) using external beam techniques. Moreover, there are recent reports of increased fibrosis and unacceptable cosmesis with APBI using external beam with BID fractionation. We adopted a once daily regimen of APBI with fractionation similar to that shown to be effective in a Canadian randomized trial of whole breast irradiation. It is unclear whether patients with DCIS or invasive lobular carcinoma (ILC) are suitable for APBI.

**Methods:**

The retrospective cohort included 310 patients with 312 tumors of T1-T2N0-N1micM0 invasive ductal carcinoma (IDC), ILC, or Tis (DCIS) treated with APBI via external beam. Most patients were treated using IMRT with 16 daily fractions of 270 cGy to a dose of 4320 cGy. The target volume included the lumpectomy cavity plus 1.0 cm to account for microscopic disease and an additional 0.5 to 1.0 cm for setup uncertainty and breathing motion. Ipsilateral breast failure (IBF) was pathologically confirmed as a local failure (LF) or an elsewhere failure (EF).

**Results:**

Median follow-up was 49 months. Among the 312 cases, 213 were IDC, 31 ILC, and 68 DCIS. Median tumor size was 1.0 cm. There were 9 IBFs (2.9%) including 5 LFs and 4 EFs. The IBF rates among patients with IDC, ILC, and DCIS were 2.4%, 3.2%, and 4.4%, respectively, with no significant difference between histologies. When patients were analyzed by the ASTRO APBI consensus statement risk groups, 32% of treated cases were considered suitable, 50% cautionary, and 18% unsuitable. The IBF rates among suitable, cautionary, and unsuitable patients were 4.0%, 2.6%, and 1.8%, respectively, with no significant difference between risk groups. Acute skin reactions were rare and long-term cosmetic outcome was very good to excellent.

**Conclusions:**

External beam APBI with once daily fractionation has a low rate of IBF consistent with other published APBI studies. The ASTRO risk stratification did not differentiate a subset of patients with a higher rate of IBF. APBI may be an appropriate treatment for women with DCIS and ILC.

## Introduction

APBI can be delivered by several techniques and has been explored to treat early stage breast cancer patients in recent years without definitive long-term data from randomized clinical trials. There are phase I/II/III trials, multi-, and single institution studies showing acceptable outcomes with limited follow-up using multicatheter brachytherapy [1-3], MammoSite brachytherapy [4-6], intraoperative radiation therapy [7-9], and more recently external beam radiation therapy via 3D conformal radiotherapy (3D-CRT) [10,11]. The last approach is the most commonly used method for APBI in the phase III NSABP B39 randomized trial with over two-thirds of patients receiving treatment with this modality.

There is no uniform agreement about which patients are suitable candidates for APBI. Many investigators limit APBI to early stage invasive ductal carcinoma (IDC) < 3 cm in size with lymph node negative disease. It is not clear whether patients with DCIS or invasive lobular carcinoma (ILC) are suitable for APBI. ASTRO has formulated a consensus statement on APBI based on limited published literature and divided patients into suitable, cautionary, and unsuitable groups [12]. Factors such as age, T stage, N stage, margin status, ER status, LVI, ductal or lobular histology, presence of pure DCIS, EIC, and multifocality/multicentricity were used to classify patients.

In the NSABP B39 trial, patients receiving APBI via external beam are treated with a dose fractionation regimen of 3.85 Gy BID for a total of 10 fractions. In a survey of our patients offering BID versus once daily fractionation, patients clearly preferred once daily treatment. Thus, we adopted a once daily regimen of 2.70 Gy for 16 fractions to a total dose of 43.2 Gy. This scheme is similar to that demonstrated by Whelan et al to be equivalent to 50.4 Gy in 1.80 Gy daily fractions in terms of efficacy and cosmesis [13]. We herein report the results of a large retrospective single institution study of once daily external beam APBI for early stage breast cancer.

## Materials and methods

### Patients

Between November 1, 2002, and June 30, 2009, 339 patients with 341 tumors of T1-T2N0-N1micM0 IDC, ILC, or Tis (DCIS) were treated with APBI via external beam at four facilities of the NorthShore University HealthSystem. Of the 339 patients, 310 patients with 312 tumors with a minimum of one year follow-up comprised the institutional review board-approved retrospective study cohort. The pretreatment patient characteristics are listed in Table 1.

**Table 1:** Pretreatment patient characteristics

Characteristic	Value
Age (years)	
Median	73
Range	42-89

Tumor size (cm)	
Median	1.0
Range	0.1-3.5

Histology	
Invasive ductal	213 (68%)
Invasive lobular	31 (10%)
DCIS	68 (22%)

Grade	
Grade 1	142 (46%)
Grade 2	114 (37%)
Grade 3	53 (17%)
Not stated	3 (1%)

Invasive tumors - EIC present	
Yes	34 (14%)
No	206 (84%)
Not stated	4 (2%)

Invasive tumors - LVI present	
Yes	5 (2%)
No	235 (96%)
Not stated	4 (2%)

DCIS tumors - Necrosis present	
Yes	36 (53%)
No	28 (41%)
Not stated	4 (6%)

Nodal Status of invasive cancer	
N0	207 (85%)
N1	2 (1%)
Nx	35 (14%)

ER/PR status	
Positive	282 (90%)
Negative	23 (7%)
Not stated	7 (2%)

HER-2/neu status of invasive cancer	
Positive	11 (5%)
Negative	231 (95%)
Not stated	2 (1%)

Surgical margins	
Negative (≥ 2 mm)	264 (85%)
Close (< 2 mm)	41 (13%)
Positive	7 (2%)

Chemotherapy	
Yes	7 (2%)
No	305 (98%)

Hormonal therapy	
Yes	166 (53%)
No	146 (47%)

*Abbreviations*: EIC = extensive intraductal component; LVI = lymphovascular invasion

### Simulation, treatment planning, and treatment

All patients underwent CT simulation in the supine position with a breast board for immobilization. CT images were acquired with 3 mm slice thickness. The clinical target volume (CTV) included the CT-defined lumpectomy cavity and surgical clips when present plus a 1.0 cm margin. The CTV was limited to 3-5 mm from the skin surface and manually edited to exclude the anterior chest wall and pectoralis muscle or, alternatively, limited to 5 mm from the lung-chest wall interface. The planning target volume (PTV) included the CTV plus a 0.5 to 1.0 cm margin to account for setup uncertainty and breathing motion. The PTV was limited to 3 mm from the skin surface.

Treatment planning was performed using either Eclipse (Varian Medical Systems, Palo Alto, CA) or Xio (CMS, St. Louis, MO) software with inverse planning-based beamlet IMRT. Field angles were similar to breast tangents with the addition of AP, lateral, and/or oblique fields. Most patients were treated with 3-5 fields in a coplanar fashion. Doses were calculated using inhomogeneity corrections. Dose-volume histograms (DVHs) were calculated for each patient. Planning guidelines were to cover the CTV with the 100% isodose line and the PTV with the 95% isodose line. The maximum hot spot was limited to 110%. Normal tissue guidelines were to limit 50% of the ipsilateral breast volume to less than 50% of the prescribed dose and 33% of the ipsilateral breast volume to less than 100% of the prescribed dose. It was recommended that the heart and lung DVHs fall below that for whole breast tangent fields.

Radiation treatments began 3 to 8 weeks after surgery. Treatment was delivered using Varian linear accelerators with 6, 10, and/or 15 MV photons. Most patients were treated with 16 fractions of 270 cGy delivered once daily five days a week for a total dose of 4320 cGy. Certain patients with superficial anteromedial lumpectomy sites were treated with en face 6 or 9 MeV electrons using 18 fractions of 250 cGy for a total dose of 4500 cGy. Bolus was occasionally used with electrons at the discretion of the treating physician.

### Follow-up

Patients were seen and examined by the treating physician 4-6 weeks after the completion of radiation and by members of the treatment team (surgical and medical oncologists) every 3-6 months for the first five years and yearly thereafter. Mammography was performed every 6-12 months. An ipsilateral breast failure (IBF) was defined as a pathologically confirmed recurrence of invasive carcinoma or DCIS in the treated breast. A recurrence was classified as a local failure (LF) if located in the treated breast quadrant or an elsewhere failure (EF) if located in a different quadrant.

### Toxicity

Acute skin toxicity was evaluated during and at the end of treatment. Cosmetic outcome was evaluated by the treating physician at follow-up visits. Toxicity was scored based on the NCI CTCAE v3.0 toxicity scale.

### Statistical methods

The follow-up time interval was calculated from the date of completion of the radiation treatments. Associations between clinical, pathologic, and treatment-related variables and clinical events were analyzed using log-rank regression. Between-group differences with respect to time-to-recurrence were depicted with Kaplan-Meier curves and using a log-rank test. A p value less than or equal to 0.05 was regarded as statistically significant. SAS 9.2 (Cary, NC) was used to carry out all statistical analyses.

## Results

The pretreatment patient characteristics are listed in Table 1. The median age at treatment was 73 with a range of 42 to 89. Most patients (68%) had IDC; 10% had ILC and 22% had DCIS. The median overall tumor size was 1.0 cm with a range of 0.1 to 3.5 cm. The tumors were grade 1 in 46%, grade 2 in 37%, and grade 3 in 17%. Of those patients with invasive cancer, 86% underwent sentinel lymph node biopsy with the vast majority (99%) being N0 and 1% having N1mic disease. Ninety percent of tumors were ER/PR positive; 53% received hormonal therapy with tamoxifen or an aromatase inhibitor. Twelve patients (4%) had HER-2/neu positive invasive tumors. Seven patients (2%) received chemotherapy.

Nintety-five percent of patients received treatment with IMRT. Thirteen patients (4%) received treatment with en face electrons due to superficial anteromedial lumpectomy sites. Two patients (1%) received treatment with 3D-CRT. Three patients had received prior whole breast irradiation (WBI) for DCIS or invasive cancer at an average of 11.3 years before treatment with APBI.

Median follow-up was 49 months with a range of 12 to 97 months. In the 312 treated breasts, there were 9 IBFs (2.9%). Of these recurrences, 5 were LFs (1.6%) that occurred at a median of 45 months after treatment. Four recurrences (1.3%) developed elsewhere in the breast and occurred at a median of 39 months after treatment. All 9 patients with recurrence underwent mastectomy. One patient with a LF subsequently developed metastatic disease and died of breast cancer 15 months later.

Three patients (1.0%) developed isolated ipsilateral axillary lymph node recurrences. Two patients underwent axillary lymph node dissection and the third had biopsy confirmation only due to advanced age and then received hormonal therapy. Four of 310 patients (1.3%) developed metastatic disease and 3 have died. The overall survival was 94.5% and the breast cancer specific survival was 99.0%. Eleven patients developed contralateral breast cancers and 21 patients developed other non-cutaneous malignancies.

**Figure 1:**
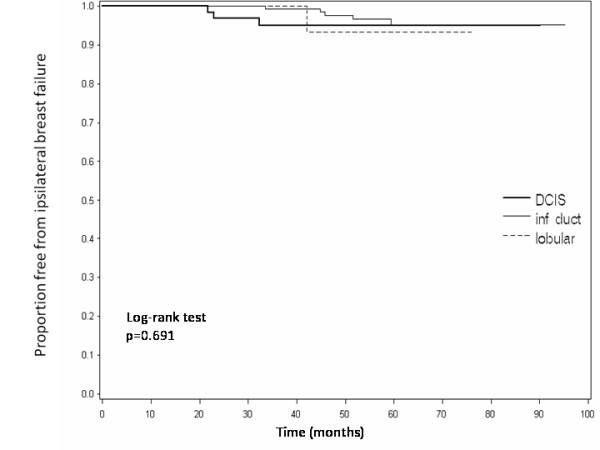
**Freedom from ipsilateral breast failure by histology vs time**.

The IBF rates according to histology are listed in Table 2. Among 213 patients with IDC whose median follow-up was 51 months, the IBF rate was 2.4%; LFs being 1.4% and EFs 0.9%. Among 31 patients with ILC whose median follow-up was 39 months, the IBF rate was 3.2% with only one EF. Among 68 patients with DCIS whose median follow-up was 48 months, the IBF rate was 4.4%; LFs being 2.9% and EFs 1.5%. There was no significant difference in IBF rates between the histologies (p = 0.691 on log-rank test, Figure 1).

**Table 2:** Treatment outcome according to histology

Histology	Number cases	Median follow-up (mos)	Ipsilateral breast failure rate	True local failure rate	Elsewhere* failure rate	Number developing metastatic disease	Number dead of breast cancer
Invasive ductal	213 (68%)	51	2.4%	1.4%	0.9%	4	3

Invasive lobular	31 (10%)	39	3.2%	0	3.2%	0	0

DCIS	68 (22%)	48	4.4%	2.9%	1.5%	0	0

* Elsewhere refers to a recurrence in the ipsilateral breast in a different quadrant than the original lumpectomy site

**Figure 2:**
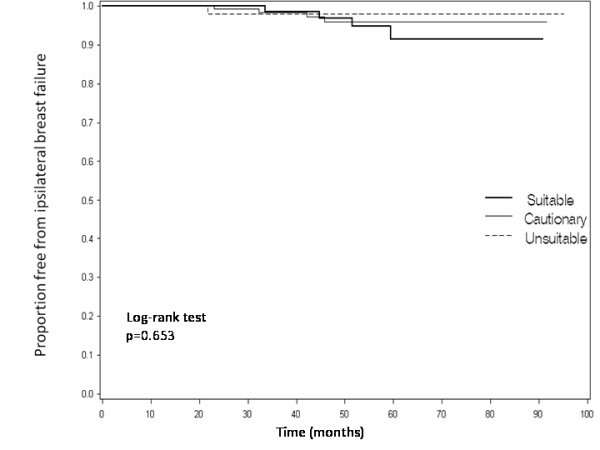
**Freedom from ipsilateral breast failure by ASTRO risk group vs time**.

The IBF rates according to ASTRO risk stratification for APBI are listed in Table 3. When patients were stratified accordingly, 32% of treated cases were considered suitable, 50% cautionary, and 18% unsuitable. The IBF rates among suitable, cautionary, and unsuitable patients were 4.0%, 2.6%, and 1.8%, respectively. There was no significant difference in IBF rates between the ASTRO risk groups (p = 0.653 on log-rank test, Figure 2). One patient in each risk group died of breast cancer.

**Table 3:** Treatment outcome by ASTRO risk stratification for APBI [12]

Risk Group	Number cases	Median follow-up (mos)	Ipsilateral breast failure rate	True local failure rate	Elsewhere* failure rate	Number developing metastatic disease	Number dead of breast cancer
Suitable	101 (32%)	52	4.0%	3.0%	1.0%	1	1

Cautionary	156 (50%)	47	2.6%	0.6%	1.9%	2	1

Unsuitable	55 (18%)	51	1.8%	1.8%	0	1	1

* Elsewhere refers to a recurrence in the ipsilateral breast in a different quadrant than the original lumpectomy site

The clinical and pathologic characteristics of patients with an IBF are detailed in Table 4. On univariate analysis among all 312 treated cases, IBF was significantly associated with ER/PR negative status (p = 0.0003). There was a trend for an association of higher grade with IBF (p = 0.085). No associations were statistically significant on multivariate analysis. For patients with IDC, ER/PR negative status was associated with IBF on univariate analysis (p = 0.02) but did not remain significant on multivariate analysis. For patients with ILC, no variables were associated with IBF on univariate analysis. For patients with DCIS, IBF was associated with ER/PR negative status (p = 0.04), presence of necrosis (p = 0.04), and positive surgical margin (p = 0.001) on univariate analysis. None of these factors was significant on multivariate analysis.

**Table 4:** Characteristics of patients with ipsilateral breast failures

#	Original histology	Age (y)	Tumor size (cm)	Margins	Grade	ER/PR	Necrosis	EIC or LVI	Hormonal therapy	Failure type	Failure histology	Time to failure (mos)
1	IDC	64	1.7	Neg	1	Pos	N/A	No	Yes	LF	IDC	45

2	IDC	65	0.6	Neg	1	Pos	N/A	No	No	LF	IDC	52

3	IDC	75	1.8	Neg	2	Pos	N/A	No	Yes	LF	IDC	60

4	IDC	77	0.9	Neg	1	Pos	N/A	No	Yes	EF	ILC	34

5	IDC	83	1.0	Close	3	Neg	N/A	No	No	EF	IDC	46

6	ILC	72	0.6	Neg	2	Pos	N/A	No	Yes	EF	IDC	43

7	DCIS	50	3.0	Pos	3	Neg	Yes	N/A	No	LF	DCIS	22

8	DCIS	65	0.9	Neg	3	Neg	Yes	N/A	Yes	LF	DCIS	23

9	DCIS	70	0.4	Neg	3	Pos	Yes	N/A	No	EF	ILC	33

*Abbreviations*: IDC = invasive ductal carcinoma; ILC = invasive lobular carcinoma; LF = local failure at lumpectomy site; EF = elsewhere failure in a different quadrant than the original lumpectomy site; EIC = extensive intraductal component; LVI = lymphovascular invasion

Acute skin reactions were rare. Grade 0, 1, and 2 skin reactions occurred in 52%, 41%, and 6% of patients, respectively. Grade 3 skin reactions occurred in 0.3% of patients and there was no grade 4 toxicity. Two patients developed symptomatic seromas requiring drainage (one during radiotherapy and one in the month following treatment). Long-term cosmetic outcome was very good to excellent in all assessed patients. There were no significant late toxicities.

## Discussion

The IBF rate of 2.9% in our 312 cases with a median follow-up of 4.1 years is similar to recurrence rates in other published studies. In RTOG 0319, 52 patients with early stage IDC received external beam APBI using 3.85 Gy BID for ten fractions [10]. The 4-year IBF rate was 6% (4% LF, 2% EF). In the ASBS MammoSite registry trial, 1,440 patients received intracavitary brachytherapy [4]. The 5-year ipsilateral recurrence rate was 3.0%. Two randomized clinical trials have investigated APBI. The TARGIT-A trial randomized 2,232 women with IDC to single fraction low energy photon intraoperative radiation or WBI [7]. The 4-year local recurrence rates were 1.20% and 0.95% in the partial breast and whole breast treatment groups, respectively. In a trial from Hungary, of 258 patients with early stage IDC randomized to either WBI or partial breast irradiation using multicatheter brachytherapy or electron beam radiation, there was no significant difference in 5-year local recurrence rates: 4.7% and 3.4% for partial and whole breast treatment groups, respectively [3].

When our data were analyzed according to the ASTRO APBI risk groups, we observed no correlation between recurrence rate and risk stratification (Figure 2). A similar finding was observed in the ASBS MammoSite registry trial [14]. The 5-year ipsilateral recurrence rates among suitable, cautionary, and unsuitable patients were 2.6%, 5.4%, and 5.3%, respectively. The conclusion of this study, like ours, was that the current ASTRO risk stratification fails to differentiate a subset of patients with a significantly higher rate of IBF when treated with APBI. A recent study from University of Wisconsin evaluated outcomes in 136 patients treated by intracavitary brachytherapy and classified as cautionary according to the ASTRO risk stratification [15]. Although 28% of patients had multiple cautionary risk factors, the IBF rate was 4.8% at 5 years. Another study from the Medical University of South Carolina with a median follow-up of 45 months also failed to demonstrate a difference in local recurrence rates by ASTRO risk stratification among 183 patients with early stage invasive cancers or DCIS when treated with intracavitary brachytherapy [16]. Although well-intentioned, rapidly evolving APBI clinical results appear to be making the ASTRO guidelines for appropriateness unduly restrictive.

There are few studies reporting outcomes of patients with DCIS treated by APBI. In our study, 68 patients had DCIS. With a median follow-up of 48 months, the IBF rate was 4.4% with 2.9% LF and 1.5% EF. Although a few factors on univariate analysis (ER/PR negative, presence of necrosis, positive surgical margin) were associated with IBF in patients with DCIS, no variable remained significant on multivariate analysis, albeit with a small number of events. In the ASBS MammoSite registry trial, 194 DCIS patients were studied [17]. At 54 months median follow-up, the IBF rate was 3.4% with 3 LFs and 3 EFs. These results are similar to those of DCIS treated with conventional WBI [18] as well as invasive cancer treated with APBI [4,10]. In a study from William Beaumont Hospital, 53 patients with DCIS were treated with APBI using MammoSite brachytherapy or external beam via 3D-CRT [19]. With a 42 month median follow-up, there was only one local recurrence. In the University of Wisconsin study, there were no ipsilateral recurrences in 32 patients with DCIS treated by intracavitary brachytherapy [15]. Although follow-up is limited with a low number of DCIS patients treated with APBI, our study and the available literature indicate that APBI may be a safe and acceptable treatment for women with DCIS.

Histopathologic studies in ILC have shown these tumors to be more multicentric than IDC [20,21]. Yet, similar to ductal cancers, most lobular cancers recur at or near the lumpectomy site [22,23]. Many studies of APBI exclude lobular histology [1,3-7,10]. Like DCIS, lobular histology is considered cautionary by the ASTRO risk stratification for APBI [12]. Furthermore, lobular histology is not included in the ABS or ASBS guidelines of appropriate patients for APBI. Not to be deterred, however, we treated 31 patients with ILC and did not observe any increased rate of local or elsewhere recurrence. Although there was a small number of 31 patients and a short median follow-up of 39 months, there was no LF and only one EF (3.2%). In a recent study from Germany of 274 women including 45 patients with ILC treated with multicatheter interstitital brachytherapy, there was no difference in local control between lobular and ductal histology with a median follow-up of 64 months [24]. Patients with ILC as well as DCIS are being accrued to the NSABP B39 randomized trial.

Our once daily dose fractionation scheme is different than that used in the NSABP B39 trial, where patients receiving external beam APBI are treated with 3.85 Gy BID for 10 fractions. However, after a survey of our patients offering BID versus once daily fractionation where patients clearly preferred once daily treatment, we adopted once daily fractionation of 2.70 Gy for 16 fractions to a dose of 43.2 Gy. This fractionation is similar to that shown by Whelan et al in a Canadian randomized clinical trial of WBI to be equivalent to 50.4 Gy in 1.80 Gy daily fractions both in terms of efficacy and cosmesis [13] and is also supported by the results of the UK START Trial B [25]. A regimen of 2.70 Gy once daily for fifteen fractions to a dose of 40.5 Gy was even used with minimal skin toxicity in a study investigating APBI and concurrent chemotherapy [26]. There are data with limited follow-up from the prospective RTOG 0319 study to support the BID NSABP B39 trial regimen [10]. However, the dose fractionation of 3.85 Gy BID for 10 fractions may be unnecessarily high for treatment of low-risk breast cancer patients [27]. There have been two cautionary reports involving the BID fractionation scheme. In a prospective study from University of Michigan involving APBI using IMRT with 3.85 Gy BID for 10 fractions, 7 of 32 patients developed unacceptable cosmesis leading to early study closure [28]. The majority of these patients had treatment plans that adhered to the dosimetric requirements of the NSABP B39 trial. In another study from Tufts University, 60 patients received APBI using 3D-CRT with 3.85 Gy BID for 10 fractions [29]. There was a high rate of moderate to severe late toxicity, especially subcutaneous fibrosis, despite adhering to the dosimetric requirements of the NSABP B39 trial.

In our study, 5 of 9 patients developed an IBF while taking hormonal therapy. It has been suggested by the CALGB 9343 randomized trial that hormonal therapy alone with tamoxifen is sufficient treatment for patients over age 70 with stage I ER-positive breast cancers [30]. With a median follow-up of 10.5 years, however, the local recurrence rate with radiation therapy and tamoxifen was significantly lowered to 2% versus 8% in the tamoxifen alone group. Along with a higher risk of local recurrence in patients treated with hormonal therapy alone, there is also the risk of side effects from administering hormonal medication for five years in an elderly patient population. There is a risk of thromboembolic disease, stroke, endometrial cancer, and hot flashes with tamoxifen. With an aromatase inhibitor, there is a risk of bone density loss with increased risk of skeletal fracture, cardiovascular disease, and symptomatic joint disorders. Moreover, patients who experience these side effects often discontinue medication early without completing the recommended five year course. In addition to being more effective than hormonal therapy alone in early stage breast cancer [31], radiation therapy has fewer and less serious side effects, and, with APBI, can be completed in a short time period rather than five years. A prospective randomized trial of APBI versus five years of an aromatase inhibitor in women over age 70 might resolve these issues.

## Abbreviations

3D-CRT: three-dimensional conformal radiation therapy; APBI: accelerated partial breast irradiation; ASTRO: American Society for Radiation Oncology; EF: elsewhere failure; IBF: ipsilateral breast failure; IDC: invasive ductal carcinoma; ILC: invasive lobular carcinoma; IMRT: intensity modulated radiation therapy; LF: local failure; WBI: whole breast irradiation

## Competing interests

The authors have no financial disclosures or conflicts of interest to report.

## Authors' contributions

AYS: Reviewed charts, collected data, and drafted the manuscript. MAL: Aided in the design of the study, reviewed the manuscript. HD: Performed statistical analyses, created figures. VTR: Aided in the design of the study, reviewed the manuscript. RKN: Aided in the design of the study, reviewed the manuscript. WDB: Aided in the design of the study, edited the manuscript. All authors read and approved the final manuscript.
